# Association of psychosocial factors with physical activity among Japanese adults aged 65 and older: a 6-year repeated cross-sectional study from the Nakanojo Study

**DOI:** 10.1186/s13030-023-00289-y

**Published:** 2023-09-12

**Authors:** Amrit Dhakal, Ken Kurisu, Sungjin Park, Kazuhiro Yoshiuchi, Yukitoshi Aoyagi

**Affiliations:** 1 Department of Stress Sciences and Psychosomatic Medicine, Graduate School of Medicine, The University of Tokyo, 7-3-1 Hongo, Bunkyo-ku, Tokyo, Japan; 2 Exercise Sciences Research Group, Tokyo Metropolitan Institute of Gerontology, 35-2 Sakaecho, Itabashi-ku, Tokyo, Japan

**Keywords:** Objective physical activity volume, Psychosocial factors, Multilevel model

## Abstract

**Background:**

Physical activity (PA) provides substantial mental and physical health benefits for individuals of all ages. A limited number of long-term or longitudinal studies have investigated the association between psychosocial factors and PA in healthy older adults aged 65 and above. This study aimed to determine the long-term relationship between psychosocial factors, such as vitality, mental health, anxiety, and depression, and objectively measure PA in older adults.

**Methods:**

Healthy participants from Nakanojo, Japan, aged 65–90, capable of walking, were included in this study and were followed up from 2008 to 2013. Those diagnosed with dementia and depression were excluded. Using a repeated cross-sectional dataset, a multilevel model was developed with psychosocial variables as independent variables and an average daily duration of PA volume of > 3 metabolic equivalents (METs) as the outcome. The Akaike information criterion was used to select the final model.

**Results:**

This study included 1108 records from 319 participants. In the multilevel model, age (coefficient = -0.106, 95% confidence interval [CI] = -0.127 to -0.086, p < 0.001) and the Hospital Anxiety and Depression Scale depression scores (coefficient = -0.019, 95% CI = -0.036 to -0.002, p = 0.026) were negatively associated with the duration of PA volume > 3 METs, whereas male sex (coefficient = 0.343, 95% CI = 0.115 to 0.571, p = 0.003) was positively associated with PA volume.

**Conclusion:**

Depressive symptoms were related to a reduced duration of PA volume of > 3 METs among these adults aged 65 and above.

## Introduction

Physical activity (PA) has substantial mental and physical health benefits by reducing the risk of chronic diseases such as cardiovascular diseases, type 2 diabetes mellitus, cancer, depression, and anxiety [1–4]. A large-scale, eight-year longitudinal study indicated that every extra 15 min of daily moderate PA, up to 100 min daily, resulted in an additional 4% decrease in mortality from any cause [5]. Increased PA can promote healthy aging and decrease demand for health and social services [6, 7]. Physical activities, like balance training, reduce the risk of falls and protect older adults from fall-related injuries [8].

World Health Organization and the United States Department of Health and Human Services published updated guidelines on the recommended amount of PA for all age groups [2, 9]. In the updated guidelines, older adults are advised to undertake a minimum of 150–300 min of moderate-intensity aerobic PA, at least 75–150 min of vigorous-intensity aerobic PA, or an equivalent combination of moderate-vigorous intensity activity throughout the week, to yield substantial health benefits [2].

Despite these recommendations, physical inactivity remains a contributor to the global health burden [10]. Notably, older adults are less inclined to meet the recommended PA thresholds due to various psychosocial factors like lack of self–efficacy [11, 12] and inadequate social support [13]. The proportion of older individuals engaging in the suggested amount of physical activities ranged from 2.4 to 83.0% across studies as per a systematic review [14].

To the best of our knowledge, only a limited number of long-term or longitudinal studies have investigated the association between psychosocial factors and PA among individuals aged 65 years and above [3, 15]. Within this demographic, long-term relationships between psychosocial factors, like the vitality (VT) and mental health (MH) subscales of the 36-Item Short Form Survey (SF-36), depression, anxiety, and PA, are less known than social support and self-efficacy [3, 12, 16]. Furthermore, only a few studies have used objective PA measurements [12], wherein the results may be influenced by recall bias in older adults [16].

Therefore, the present study used a longitudinal dataset to determine the long-term (six years) association of psychosocial factors with objectively measured PA volume of > 3 metabolic equivalents (METs) in adults aged 65 years and older.

## Methods

### Participants and study design

Healthy residents of Nakanojo town, aged 65–90 years and capable of walking, were included. They were followed from 2008 to 2013. Participants were recruited through flyers distributed during health checkups to residents of Nakanojo, a medium-sized town located 150 km northwest of Tokyo. We excluded the records of participants with dementia or depression at baseline and throughout the follow-up period of six years. The diagnosis was ascertained by questionnaires reported by either the participants or their families. The question asked if the participants’ treating doctors had examined them and informed them of these conditions, either within the past year or more than a year prior to their participation in this study. We did not exclude diseases other than dementia and depression because this study basically recruited participants capable of walking.

Recordings of daily PA were commenced following an individual’s acceptance for participation in the study. Sociodemographic factors, living conditions, and questionnaires concerning psychosocial status were administered every July for six years [3]. In this study, we used PA volume and other variables measured in the same year, meaning the outcome variables, i.e., PA data, were measured before or after independent variables, resulting in a repeated-measures cross-sectional design.

### Measurements

#### Sociodemographic factors

Sociodemographic factors such as age, sex, smoking status, alcohol intake status, marital status, and living conditions (living alone or not) were measured.

#### Physical activity measurement

Every four seconds, an electronic accelerometer with a 36-day storage capacity (modified Kenz Lifecorder from Suzuken Co., Ltd., Nagoya, Japan) supplied readings of the PA volume [3, 16]. It was fastened to a waist belt on the left side of the participant’s body. Data collected during the waking hours were examined using an arbitrary computer program before the calculation of scores. The participants wore the device during each 24-hour period from midnight to the following midnight throughout the follow-up period. This identified continuous intervals that indicated the removal of the accelerometer for activities such as bathing, taking a nap, or dressing. Data from these intervals were excluded from the analysis. The average daily duration of PA volume levels > 3 METs was derived from these records.

#### Short-form health survey (SF-36) questionnaire

The VT and MH of SF-36 were assessed using a validated Japanese-language version of the 36-item Short Form Health Survey (SF-36) questionnaire [17]. This questionnaire includes 35 questions about the previous month and measures eight different aspects of health status. The VT and MH scores for each subscale range from 0 to 100.

#### Hospital anxiety and depression scale measurement

The Hospital Anxiety and Depression Scale (HADS) scores measure psychological maladjustment of an individual. It is a 14-item scale with seven items each for the anxiety and depression subscales. A validated Japanese translation of HADS was used for this study [18].

### Statistical analysis

The normality of PA volume was checked using the Kolmogorov-Smirnov (K-S) test. When the value was not distributed normally, a natural log transformation was performed, and normality was rechecked.

Pearson correlations between continuous variables and raw or log-transformed PA volumes were quantified. The comparison of raw or log-transformed PA volumes among the components of categorical variables was performed using the independent t-test or one-way ANOVA, according to the number of components and variance homogeneity.

Multilevel modeling was used because this study used data with a hierarchical structure in which multiple records from each participant were analyzed [19, 20]. Models were developed, including a null intercept model, and the fixed and random effects of level 1 and level 2 predictor variables were examined. The Akaike information criterion (AIC) was used to select the best model fit. Independent variables included age, sex, and psychosocial variables that exhibited a significant correlation with PA volume. The dependent variable was the average daily duration of PA volume at a level greater than 3 METs.

A significance level of 5% was set (p < 0.05). The analyses were conducted using SPSS version 28.0.1.1 and R version 4.2.0.

## Results

### Descriptive statistics

A total of 319 participants were included in this study. Descriptive statistics are presented in Table 1.

**Table 1:** Descriptive statistics

	N = 319
Age at baseline (in years)	
Mean (SD)	75.17 (4.74)
Median (IQR)	75.00 (6)
Sex, N (%)	
Male	159 (49.8)
Female	160 (50.2)
Smoking status, N (%)	
Smokes or smoked in the past	75 (23.5)
Does not smoke	238 (74.6)
NA	6 (1.9)
Alcohol, N (%)	
Drinks almost everyday	72 (22.6)
Drinks occasionally	84 (26.3)
No or rarely	161 (50.5)
NA	2 (0.6)
Living alone, N (%)	
Yes	43 (13.5)
No	271 (85.0)
NA	5 (1.6)
Marital status, N (%)	
Living together	233 (73.0)
Separated	6 (1.9)
Divorced	2 (0.6)
Spouse deceased	64 (20.1)
NA	14 (4.4)
BMI, kg/m^2^	
Mean (SD)	22.73 (2.73)
Median (IQR)	22.64 (3.74)
Vitality	
Mean (SD)	71.28 (18.22)
Median (IQR)	75.00 (18.75)
MH	
Mean (SD)	78.55 (17.56)
Median (IQR)	85.00 (28.75)
HADS anxiety score	
Mean (SD)	3.96 (3.03)
Median (IQR)	3.00 (4)
HADS depression score	
Mean (SD)	3.72 (3.29)
Median (IQR)	3.00 (4)
HADS total score	
Mean (SD)	7.56 (5.68)
Median (IQR)	6.00 (8)

BMI, body mass index; HADS, Hospital Anxiety and Depression Scale; IQR, interquartile range; SD, standard deviation; NA, not available

Because the raw PA volume data did not exhibit a normal distribution in the K-S test (D = 0.195, P < 0.001), log-transformed values of PA volume were used instead. The K-S test showed that the values were normally distributed (D = 0.068, P = 0.106). The mean PA volume for log-transformed was 2.32 (SD, 1.17).

Table 2 shows the Pearson correlation between continuous variables and the log-transformed PA volume. Several variables were significantly associated with the log-transformed PA volume, including age, VT, and HADS depression scores.

**Table 2:** Pearson Correlation between continuous variables and the log-transformed PA intensity

	Correlation (95% CI)	P-value
Age	-0.314 (-0.409 to -0.211)	**< 0.001**
BMI	0.011 (-0.101 to 0.123)	0.846
VT	0.182 (0.072 to 0.287)	**0.001**
MH	0.080 (-0.032 to 0.190)	0.161
HADS Anxiety	-0.062 (-0.175 to 0.053)	0.293
HADS Depression	-0.121 (-0.232 – -0.007)	**0.038**
HADS Total	-0.071 (-0.187 – 0.046)	0.233

BMI, body mass index; VT, vitality; MH, mental health; HADS, hospital anxiety and depression scale

No significant differences were observed in log-transformed PA volumes among each category of binary variables, such as sex, when compared using the Student’s t-test. Equal variances were assumed, which were tested by F-test and its significance level. The details are depicted in Table 3.

**Table 3:** Relations of categorical variables with log-transformed PA volume, at baseline (independent t-test)

	Log transformed Intensity, mean (SD)	F-test	P-value	t value	P-value
Sex		1.789	0.182	0.531	0.596
Male	2.424 (1.213)				
Female	2.357 (1.129)				
Smoking		0.420	0.517	0.591	0.555
Smokes/smoked	2.451(1.167)				
Does not smoke	2.363(1.116)				
Living alone		2.267	0.133	-1.061	0.290
Yes	2.219(1.229)				
No	2.414(1.107)				

Furthermore, there were no significant differences in log-transformed PA intensities among each category with more than two components when compared using the one-way ANOVA test. The F-value represents the variances between group means. The results from the tests are depicted in Table 4.

**Table 4:** One Way ANOVA test for categorical variables with more than two components

	Log transformed PA volume, mean (SD)	F value	P-value
Alcohol		1.084	0.349
Drinks almost everyday	2.440(1.121)		
Drinks occasionally	2.518(1.111)		
No or rarely	2.304(1.135)		
Marital status		2.483	0.061
Living together	2.476(1.103)		
Separated	2.079(1.293)		
Divorced	1.257(1.129)		
Spouse deceased	2.133(1.086)		

### Multilevel modeling

After excluding 20 records with depression and 6 records with dementia, a total of 1108 records were available from 319 participants. Records with missing values for age, sex, and significantly correlated psychosocial variables of interest (i.e., VT and HADS depression scores) were excluded. The level 1 and level 2 equations in the multilevel model selected according to the AIC are as follows:

Level 1 equation:

1 $$\log \left( {PA{\text{ }}volume} \right)_{ij} = {\text{ }}\Pi_{0i}{\text{ }} + {\text{ }}\Pi_{1i}\Delta Age_{ij} + \Pi_{2i}HADS(D)_{ij} + \varepsilon_{ij}$$

Level 2 equation:

2 $$\Pi_{0i} = \gamma_{00} + {\text{ }}\gamma_{01}Sex_i + \zeta_{0i}$$

3 $$\Pi_{1i} = \gamma_{10} + \zeta_{1i}$$

4 $$\Pi_{2i} = \gamma_{20} + \zeta_{2i}$$

Table 5 lists the model coefficients. The coefficient of the HADS depression score (HADS-D) was found to be statistically significant but small (coefficient = -0.019; 95% CI = -0.036 to -0.002; p = 0.026). Age, a differential value of age from the participants’ average, and being male were statistically significant, with coefficients of -0.106 (95% CI = -0.127 to -0.086; p < 0.001) and 0.343 (95% CI = 0.115 to 0.571; p = 0.003), respectively.

**Table 5:** Mean effects of age, HADS depression score, and sex for log-transformed PA volume in the multilevel model

	Mean (95% CI) effect	P value
Intercept	2.470 (2.308 to 2.632)	< 0.001
ΔAge (years)	-0.106 (-0.127 to -0.086)	< 0.001
HADS-D	-0.019 (-0.036 to -0.002)	0.026
Male sex	0.343 (0.115 to 0.571)	0.003

Δ, differential value from mean; HADS-D, depression subscale of hospital anxiety and depression scale

The intercept represents the mean log-transformed PA volume for female participants with a ΔAge of 0 and a HADS depression score of 0. Because VT was attempted in the multilevel model development but did not improve AIC, it was not included in the final model.

## Discussion

To the best of our knowledge, this is the first study involving healthy older adults for a duration of six years that employs objective PA measurements and examines the association between various psychosocial factors, like vitality and mental health, using the SF-36 questionnaire, depression, anxiety, and PA, rather than focusing on a single psychosocial factor. In the multilevel model developed using the longitudinal repeated cross-sectional dataset, increasing age and depression score predicted a lower PA volume, and being male was positively related to a PA volume of > 3 METs, compared to females.

This study showed a significant negative, albeit weak, correlation between depression scores and PA volume. In this study, the measurement of psychosocial variables did not consistently precede the measurement of PA volume. Consequently, owing to the cross-sectional design of this study, a causal relationship could not be determined. Nonetheless, this finding is in line with that of another longitudinal study that revealed a significant correlation between depression and PA in older adults [3].

A cross-sectional study conducted by Yoshiuchi et al. [16] using an accelerometer for a year and another by Harris et al. [11] using an accelerometer for only a week also support this study’s findings. Notably, these studies showed a stronger correlation than the present study. However, these studies included participants with depression and did not exclude those with dementia. In contrast, the present study excluded participants who were diagnosed with either depression or dementia. This difference in exclusion criteria may have resulted in the heterogeneity of the results. Namely, the weak correlation in the present study, compared to the previous studies, may be attributable to the exclusion of patients with these two conditions, who are generally less active than healthy individuals. A stronger correlation might have been observed had we included these patients, as was done in earlier studies. Future longitudinal studies should compare participants with and without depression using objective PA measurements.

This study has several limitations. First, we cannot determine causality for any relationships due to the cross-sectional design. Second, our research focused on a single practice in a medium-sized town in Japan, which limits the generalizability of our findings to urban areas with larger populations. Third, the analysis did not include other potential confounding factors like personality traits and education level. Finally, the participants were generally healthy, and the findings may not apply to clinical settings.

In conclusion, depressive symptoms were significantly negatively correlated with PA volume in healthy older adults. To explore causality, the relationship between depressive symptoms and PA volume among older adults, particularly those aged 65 and above, should be further explored in urban and rural areas through additional longitudinal studies.

## Declarations

### Data sharing

We cannot share the data due to The University of Tokyo data sharing policy.

## Abbreviations

AIC: Akaike information criteria
HADS: Hospital Anxiety and Depression Scale
K-S test: Kolmogorov-smirnov test
MH: mental health
PA: physical activity
SD: standard deviation
SF-36: Short Form-36
VT: vitality
